# Protoporphyrin (PPIX) efflux by the MacAB-TolC pump in *Escherichia coli*

**DOI:** 10.1002/mbo3.203

**Published:** 2014-09-26

**Authors:** Evelyne Turlin, Gesine Heuck, Maria Inês Simões Brandão, Noémie Szili, J R Mellin, Norbert Lange, Cécile Wandersman

**Affiliations:** 1 Unité des Membranes Bactériennes, Département de Microbiologie, Institut Pasteur75724, Paris Cedex 15, France; 2 School of Pharmaceutical Sciences, Université de Genève, University of Lausanne30 Quai Ernest Anserment, CH-1211, Genève 4, Suisse

**Keywords:** Heme homeostasy, iron chelation, MacAB-TolC pump, photosensitivity, protoporphyrin IX

## Abstract

In most organisms, heme biosynthesis is strictly controlled so as to avoid heme and heme precursor accumulation, which is toxic. *Escherichia coli* regulates heme biosynthesis by a feedback loop involving heme-induced proteolytic cleavage of HemA, glutamyl-tRNA reductase, which is the first enzyme in the heme biosynthetic pathway. We show here that heme homeostasis can be disrupted by overproduction of YfeX, a cytoplasmic protein that captures iron from heme that we named deferrochelatase. We also show that it is disrupted by iron chelation, which reduces the intracellular iron concentration necessary for loading iron into protoporphyrin IX (PPIX, the immediate heme precursor). In both cases, we established that there is an increased PPIX concentration and we demonstrate that this compound is expelled by the MacAB-TolC pump, an efflux pump involved in *E. coli* and *Salmonella* for macrolide efflux. The *E. coli macAB* and *tolC* mutants accumulate PPIX and are sensitive to photo-inactivation. The MacAB-TolC pump is required for *Salmonella typhimurium* survival in macrophages. We propose that PPIX is an endogenous substrate of the MacAB-TolC pump in *E. coli* and *S. typhimurium* and that this compound is produced inside bacteria when natural heme homeostasis is disrupted by iron shortage, as happens when bacteria invade the mammalian host.

## Introduction

Heme is the major iron-containing molecule in vertebrates. Bacteria invading such hosts have systems for acquiring heme from host hemoproteins, for transporting heme and for retrieving its iron (Wandersman and Delepelaire 2012). A major class of proteins involved in extracting iron from heme is that of the heme oxygenases, which cleave the tetrapyrrole ring of the heme molecule, thereby releasing the bound iron atom along with biliverdin and carbon monoxide (Frankenberg-Dinkel 2004). A second major class is comprised of several Dyp peroxidases, identified in *Escherichia coli* (YfeX and EfeB) (Letoffe et al. 2009) and *Staphylococcus aureus* (FepB) (Turlin et al. 2013), which facilitate the release of iron from heme in a manner which preserves the tetrapyrrole ring, generating a free iron cation and protoporphyrin IX (PPIX). Either YfeX or EfeB is required for the use of exogenous heme as an iron source in *E. coli* strains equipped with heme uptake systems. While FepB and EfeB operate in the extracytoplasmic compartment, YfeX is found in the cytoplasm. Overproduction of YfeX leads to an increased intracellular pool of PPIX, which is potentially toxic in the presence of light and oxygen, as high PPIX levels result in production of reactive oxygen species (ROS) (Kochevar 1987).

In addition to PPIX generated by extraction of iron from heme by Dyp peroxidases, PPIX can also be formed during heme biosynthesis. In *E. coli*, the first enzyme in heme biosynthesis, HemA, catalyzes formation of glutamyl-tRNA. This intermediate compound is then converted by HemL into 5-aminolevulinic acid (5-ALA). The condensation of two 5-ALA molecules yields porphobilinogen. Subsequently, four porphobilinogen molecules condense to form uroporphyrinogen III which is converted into coproporphyrinogen III. The molecule give coproporphyrin III named COPIII. Finally, decarboxylation and oxidation of COPIII lead to the formation of PPIX. The last step in heme biosynthesis is accomplished by insertion of Fe^++^ into PPIX, which, in prokaryotes, is catalyzed by the ferrochelatase HemH (Panek and O'Brian 2002). In most organisms, enzymes involved in heme biosynthesis are tightly controlled to prevent porphyrin accumulation. *Salmonella* regulates heme biosynthesis through a negative feedback loop involving heme-induced proteolytic degradation of HemA (Schobert and Jahn 2002). However, heme homeostasis might be disrupted in an iron-depleted environment such as the mammalian host, with the availability of iron being too limited to allow ferrochelatase-mediated iron insertion into PPIX. In the absence of heme, feedback inhibition of HemA would not occur. However, such loss of feedback inhibition has not been studied. Heme homeostasis can also be intentionally disrupted by supplementing a bacterial culture with 5-ALA, the first precursor in heme biosynthesis. This bypasses the negative feedback inhibitory mechanism controlling heme production and results in overproduction of a number of heme intermediates, but primarily COPIII, which may represent up to 94% of intracellular porphyrins in the presence of 5-ALA (Verderber et al. 1997; Tatsumi and Wachi 2008).

Nonetheless, there is only a slight accumulation of porphyrin inside cells, suggesting the existence of a mechanism to maintain porphyrin homeostasis. In the presence of exogenous 5-ALA, a *tolC* mutant accumulates COPIII in the cells (Tatsumi and Wachi 2008). TolC is an outer membrane protein which interacts with several inner membrane efflux pumps to extrude exogenous xenobiotic compounds and intracellular metabolites (Nikaido 2009; Horiyama et al. 2010; Rosner and Martin 2013). The fact that the *tolC* mutant accumulates porphyrins led to the hypothesis that TolC might be involved in porphyrin efflux in *E. coli*.

In Gram-negative bacteria, efflux pumps have a tripartite organization with an outer membrane protein belonging to the TolC channel–tunnel family, a periplasmic membrane fusion protein and an inner membrane transporter (Nikaido and Pages 2012). The bacterial transporters are divided into five classes of transporters: small multidrug resistance (SMR), major facilitator superfamily (MFS), resistance nodulation division superfamily (RND), multidrug and toxic compound extrusion (MATE), and ATP binding cassette (ABC) transporters. While the first four transporters utilize energy derived from a proton gradient, the ABC transporters are driven by ATP hydrolysis (Nikaido 2009). ABC transporters have been implicated in multidrug resistance in cancer cells, conferring resistance to chemotherapeutic drugs (Higgins 2001). However, their presence and involvement in drug efflux is less important in bacteria. Although more than 16 proton-dependent pumps have been identified in *E. coli*, only one ABC-type tripartite efflux transporter has been characterized. This pump, named MacAB-TolC, confers macrolide-specific resistance when overexpressed (Kobayashi et al. 2001). The MacAB-TolC pump has also involved in enterotoxin secretion in *E. coli* (Yamanaka et al. 2008). The MacB ortholog (PvdT) is involved in efflux of the pyoverdin siderophore (Imperi et al. 2009).

In this study, we investigate the mechanism of PPIX efflux and we report that the MacAB-TolC pump is a major pump involved in efflux of PPIX.

## Experimental Procedures

### Bacterial strains and plasmids

*Escherichia coli* strains used in this study are listed in Table 1. *Escherichia coli* K-12 strain JP313 and C600 were from laboratory collections. Strains JP313 *ΔentF::Cm* and JP313 *ΔmacAB::Km* were constructed in the present work (see below). Strains JP313 *ΔtolC::Tn10*, JP313 *ΔentF::Cm ΔtolC*:*:Tn10*, and JP313 *ΔentF::Cm*, *ΔmacAB::Km*) were constructed by P1 transduction of *ΔtolC::*Tn10 from SL767 or *ΔmacAB::Km* from JP313 *ΔmacAB::Km*. Strain JP331*ΔacrAB::Km* was also constructed by P1 transduction of *ΔacrAB::Kan* from AG100A (Okusu et al. 1996). All mutations were introduced and studied in an isogenic background, JP313. To simplify reading, they were named in results and tables only by their relevant mutations.

**Table 1:** Bacterial strains and plasmids.

Name	Specification	Source
*Escherichia coli* strains		
JP313 (WT)	araD139 ΔlacU169 LAM-flhD5301 fruA25relA1 rpsL150 (str^R^) rbsR22 deoC1 araΔ714	Pogliano et al. (1997)
SL767	C600*tolC::Tn10*	Laboratory collection
AG100A	AG1 Δ*acrAB::Kan*	Okusu et al. (1996)
*acrAB*	JP313Δ*acrAB::Km*	This work
*entF*	JP313Δ*entF::cm*	This work
*macAB*	JP313Δ*macAB::Km*	This work
*tolC*	JP313Δ*tolC::Tn10*	This work
*entF tolC*	JP313Δ*entF::cm*, *tolC::Tn10*	This work
*entF macAB*	JP313Δ*entF::cm*, Δ*macAB::Km*	This work
Plasmids		
pBAD24	Expression vector. Amp^R^	Guzman et al. (1995)
(pBAD24-yfeX)	pBAD24 vector carrying the *6His-yfeX* gene under p*ara* promoter	Turlin et al. (2013)
pBAD33	Expression vector. Cm^R^	Guzman et al. (1995)
(pBAD33-macAB)	pBAD33 vector carrying the *macAB* genes under p*ara* promoter	This work
(pBAD33-acrAB)	pBAD33 vector carrying the *acrAB* genes under p*ara* promoter	This work
(pBAD33-acrEF)	pBAD33 vector carrying the *acrEF* genes under p*ara* promoter	This work
(pBAD33-mdtEF)	pBAD33 vector carrying the *mdtEF* genes under p*ara* promoter	This work
(pBAD33-EmrAB)	pBAD33 vector carrying the *emrAB* genes under p*ara* promoter	This work
(pACYC184-tolC)	pACYC184 vector carrying tolC gene under ptet promoter	Masi and Wandersman

All mutations were introduced and studied in an isogenic background, JP313. To simplify reading, they were named in results and tables only by their relevant mutations. Plasmids were named only by their gene insert. Plasmids without insert by the name of the void plasmid.

pBAD24, pBAD24-6His-yfeX (pBAD24-yfeX), pBAD33, and pACYC84-tolC are from the laboratory collection. pBAD33-macAB, pBAD33-acrAB, pBAD33-acrEF, pBAD33-mdtEF, and pBAD33-emrAB were constructed in the study. pBAD24 and pBAD33 are compatible the first with colE1 and the second with pACYC replication origins. The first carries Amp and the second Cm resistance genes.

### Media and growth conditions

Bacteria were grown aerobically at 37°C or 30°C in LB medium. For arabinose induction, 0.2% l-arabinose (ara) was added to induce the p*ara* promoter. When required, 2,2′dipyridyl (Dip) was added to a final concentration of 100 *μ*mol/L and 5-ALA at a concentration of 10 *μ*g mL^−1^. Cells were grown overnight in LB medium and 100-fold-diluted in the morning. When they reached an OD_600_ of 0.5, Dip was added at the indicated concentrations. Cells were harvested when growth stopped. When necessary, antibiotics were added at the following concentrations: ampicillin, 100 *μ*g mL^−1^; kanamycin, 50 *μ*g mL^−1^; chloramphenicol, 15 *μ*g mL^−1^; tetracycline, 10 *μ*g mL^−1^.

### Genetic and molecular biology techniques

Mutant *ΔmacAB::Kan* was constructed by replacing the entire *macAB* operon, with an antibiotic cassette using the CF10230 strain, as previously described (Mechold et al. 2006). This mutation was subsequently transduced into JP313 strain using the P1vir phage. Verification of deletions was done by PCR.

Plasmids carrying *acrAB, acrEF, macAB, emrAB*, *and mdtEF* genes were constructed by amplification of *E. coli* MG1655 genomic DNA using complementary oligonucleotides. Amplified fragments were inserted into pBAD33 digested with SacI or KpnI and XbaI, giving plasmids pBAD33-acrAB, pBAD33-acrEF, pBAD33-macAB, pBAD33-emrAB, and pBAD33-mdtEF carrying the corresponding genes under the p*ara* promoter and regulated by by AraC in the presence of arabinose. Full induction was obtained by addition of 0.2% arabinose to the medium.

All plasmids used in this study are listed in Table 1. Oligonucleotides used for each cloning are available on demand.

### Electrophoresis and western blot

**Figure 1:**
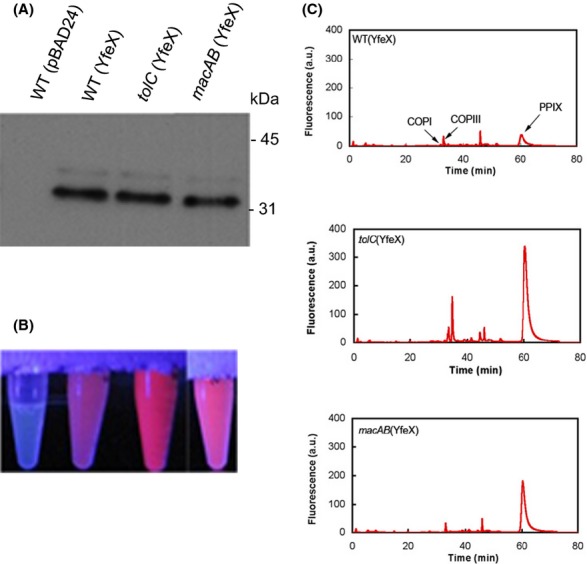
Accumulation of Protoporphyrin IX (PPIX) in tolC and macAB mutants. (A) Immunodetection of YfeX amounts in various strains 10 indicated above the western blot. Protein extracts were obtained as described in Experimental Procedures (porphyrin extraction and HPLC analysis). Levels of 6His-YfeX were detected by immunoblotting using anti-His antibodies. Each lane was loaded with 10 lL of a cell extract of cultures grown to an OD600 of 1. Each extract contained the same total protein concentration of 2 mg mL^−1^ as measured by Bradford. (B) Visualization of the fluorescence of soluble fraction under near-UV light. The name of the corresponding culture strain is indicated above the western blot. (C) HPLC fluorescence chromatograms of porphyrins. The fluorescent soluble fraction of strains WT and mutants indicated in the figure were prepared as described in Experimental Procedures. The extracted porphyrin were separated by HPLC. The retention positions of control porphyrins (COPI, COP III, and PPIX) are indicated on the graph by arrows. There are not many endogenous fluorophors except prophyrins that can be excited at 405 nm and do fluorescence at 635 nm (chlorins and chlorophylls are fluorescing at about 670 nm when excited at 400 nm). The mass spectrometry data of the various fluorescent compounds were performed in Letoffe et al. 2009.

Sodium dodecyl sulfate polyacrylamide gel electrophoresis (SDS-PAGE) and western blot were carried out according to standard protocols. Cultures were grown to an OD_600_ of 1. Cells were harvested by centrifugation for 20 min at 5000*g* at 4°C. The cell pellet was resuspended in 50 mmol/L Tris-HCl pH 8.0, 0.3 mol/L NaCl to OD_600_ of 50/mL and disrupted by sonication, then centrifuged at 20,000*g* for 60 min at 4°C to remove cellular debris. Protein concentration in cell extracts was determined by Bradford protein assay and is indicated in the Figure 1. Ten microliters of aliquots were loaded on SDS PAGE. Bound antibodies were detected with secondary anti-rabbit antibodies coupled with horseradish peroxidase and revealed by chemiluminescence.

### Porphyrin extraction and HPLC analysis

For quantitation of porphyrin content in cells, bacteria were grown overnight in LB broth supplemented with 100 *μ*g mL^−1^ ampicillin. In the morning, cultures were diluted 1:100 in fresh media supplemented with the appropriate antibiotics in either the presence or absence of arabinose to induce YfeX overproduction. Cultures were grown to an OD_600_ of 1. Cells were harvested by centrifugation for 20 min at 5000*g* at 4°C. The cell pellet was resuspended in 50 mmol/L Tris-HCl pH 8.0, 0.3 mol/L NaCl to OD_600_ of 50/mL and disrupted by sonication, then centrifuged at 20,000*g* for 60 min at 4°C to remove cellular debris. Porphyrin extraction was performed as described by Létoffé et al. Briefly, to extract the porphyrins from soluble fractions, 800 *μ*L of extraction solvent (ethanol/Dimethyl Sulfoxide/acetic acid; 80/20/1; v/v/v) were added to 200 *μ*L of the supernatant. The mixtures were sonicated in 5 cycles of 5 sec at 0°C, amplitude 10%, using a sonicator probe (Branson Digital Sonifier, Danbury, CT, USA). The samples were centrifuged at 12,500*g* for 5 min and porphyrin-containing supernatants were used for analysis. Complete extraction of porphyrins from the sample was verified by the absence of porphyrin fluorescence in the pellet. Porphyrins were separated by high-performance liquid chromatography (HPLC) (LaChrom; Merck, Darmstadt, Germany) as described previously using a 125/4 Nucleodur C18 gravity 3 *μ*m column (Macherey-Nagel, Oensingen, Switzerland) with the corresponding precolumn. An elution gradient was run over 80 min with solvent A (acetate buffer pH5.1, 0.5 mol/L/acetonitrile; 90:10; v/v) and solvent B (methanol/acetonitrile; 90:10; v/v). The flow rate was 1 mL min^−1^. Porphyrins were excited at 405 nm and measured at 620 nm using a LaChrom L-7480 fluorescence detector (Merck). Porphyrin peaks were identified by comparison with a chromatogram of porphyrin standard solutions (Frontier Scientific, Carnforth, UK). The HPLC solvent gradient was adjusted so that the retention time increased with the lipophilicity of the analyte.

### Determination of minimal inhibitory concentration

The antibiotic minimal inhibitory concentration (MIC) was determined following the procedure described in (Kobayashi et al. 2001). Briefly, bacteria harboring the various plasmids were grown at 37°C to an OD_600_ of 1 and poured onto LB plates. Determination of the MIC of erythromycin (ERY) was done in triplicate by the twofold dilution method on LB plates seeded with a 10 *μ*L spot of bacterial dilution containing 10^4^ colony forming unit (CFU). After 12 h of growth at 37°C, the MIC was determined as the MIC in *μ*g mL^−1^ that completely inhibited colony formation.

### Drugs susceptibility

Overnight LB cultures of JP313ΔacrAB strains harboring the various plasmids used were grown at 37°C and then appropriately diluted. Antibiograms were done using discs from BioRad (Bio-Rad, Hercules, CA, USA) charged with kanamycin as a strain marker, ampicillin and chloramphenicol as markers of the plasmid vectors, and various hydrophobic drugs: ERY as a representative of 14-membered macrolides, azithromycin (AZI) as a representative of 15-membered macrolides, and novobiocin (NOV), trimethoprim (TMP), and desoxycholate (DOC). Mueller–Hinton (BioRad) agar plates were incubated for 24 h at 37°C and diameters of inhibition zones around drug-containing discs were measured manually.

### Porphyrin light sensitivity tests

Photosensitivity test experiments were carried out with JP313 and its isogenic mutants *tolC* and *macAB* carrying either pBAD24 or pBAD24-6His-yfeX. Bacteria were cultured overnight and diluted 100-fold in the morning. When cultures reached mid-log level growth, they were diluted again into Phosphate Buffered Saline (PBS) to obtain a final OD_600_ of 0.0001. About 2.5 mL of each suspension was aliquoted into 6-well polystyrene plates (diameter 35 mm) and placed on a light table equipped with Narwa LT 18W/O18 light tubes (PCI Biotech, Oslo, Norway). The radiation intensity was 1.375 mW/cm^2^. Cells were exposed to light for up to 4 h. Aliquots were taken at the corresponding time points, serially diluted and cell survival was assessed by counting the number of CFUs in light-exposed and unexposed samples.

## Results

### *Escherichia coli* tolC mutant accumulates PPIX

In the presence of exogenously provided 5-ALA, wild type (WT) as well as *tolC* mutants formed mainly COPIII (>94% of total intracellular porphyrins). However, while the the WT strain expelled this COPIII, high amounts of intracellular COPIII were found in the *tolC* mutant (Tatsumi and Wachi 2008). To determine whether TolC is involved in PPIX efflux, strains WT (pBAD24), WT (pBAD24-yfeX), and *tolC* (pBAD24-yfeX) were grown with arabinose to induce *yfeX*. Immunodetection with anti-His antibodies showed that both strains (*tolC+* and *tolC*) produced the same amounts of YfeX under arabinose induction (Fig. 1A). Both of them showed characteristic red porphyrin fluorescence under UV light irradiation upon YfeX induction. However, the fluorescence intensity of the *tolC* mutant was considerably higher (Fig. 1B). The observation was reversible by complementation with a plasmid expressing *tolC* pACYC184-tolC which reduced the fluorescence to the level of the *tolC+* strain (Fig. S1).

To determine whether the observed fluorescence corresponded to increased accumulation of PPIX, we examined cells by fluorescence HPLC. Cells were grown in LB broth to OD_600_ of 1 in the presence of arabinose to induce *yfeX* overexpression. Cells were collected by centrifugation and porphyrins were extracted from soluble fractions by solvent treatment. Figure 1C shows that both strains *tolC+* (pBAD24-yfeX) and *tolC* (pBAD24-yfeX) contained PPIX. However, the *tolC* mutant accumulated 9 times more PPIX than the corresponding *tolC+* strain.

In the absence of y*feX* induction, the COPI, COPIII, and PPIX levels were also higher in the *tolC* mutant than in the *tolC*+ strain (data not shown). This suggests that the *tolC* mutant is impaired in its ability to expel a number of intermediates generated during normal heme biosynthesis. When *yfeX* is induced, PPIX efflux requires TolC and most likely also inner membrane efflux pumps.

In *E. coli*, the TolC protein is shared by several pumps to expel drugs. Twenty transporters (11 MF, 2 SMR, 6 RND, and 1 ABC) were able to confer drug resistance when overproduced, and several had redundant functions (Nishino and Yamaguchi 2001). In the case of COPIII, which is the major porphyrin formed when cells are grown in the presence of 5-ALA, Tatsumi and Wachi (2008) reported that inactivation of the individual pumps AcrAB, AcrD, AcrEF, EmrAB, MacAB, MdtEF, MdtABC, and EmrY did not further increase COPIII accumulation, suggesting that these pumps might have redundant functions for COPIII efflux, or that COPIII is expelled by other pumps. We hypothesized that PPIX efflux pumps might also be redundant and that single inactivation would not decrease PPIX efflux.

### Competition studies between PPIX and antibiotics

In an attempt to identify pumps involved in PPIX efflux, we tested whether YfeX-driven overproduction of PPIX might affect the efflux capacities of various tripartite pumps. The following pump genes were cloned on plasmid pBAD33: *acrAB, mdtEF, acrEF, emrAB*, and *macAB,* leading to protein pump overexpression upon arabinose induction. To measure the effects that pump overproduction produced on drug resistance phenotypes, the constructed plasmids were introduced into *acrAB* mutant, which is very sensitive to drugs due to the loss of the major efflux pump (AcrAB). Overexpression of all pumps tested resulted in increased MICs of strain *acrAB* for several drugs such as NOV, azythromycin (Fig. S2), and ERY (Table 2), in agreement with previously published results(Nishino and Yamaguchi 2001). We next introduced pBAD24-yfeX into *acrAB mutant,* also expressing the various pumps. Upon arabinose induction, all strains were fluorescent (data not shown). YfeX production was determined by western blot and showed similar levels of the protein in the various strains (data not shown). YfeX-driven PPIX overproduction failed to change the ERY MIC values of *acrAB* strain carrying acrAB, mdtEF, acrEF, and emrAB on pBAD33 (Table 2). In contrast, PPIX production reduced the ERY MIC value of *acrAB* (macAB) to the level of the *acrAB* mutant (Table 2). Excessive PPIX production also reduced the MIC of other tested antibiotics (azythromycin, NOV, and deoxycholate) expelled by the MacAB-TolC pump (Fig. S2). Since MacAB is the only pump strongly inhibited by PPIX overproduction, it is unlikely that PPIX inhibits physiological function required for drug efflux. It is more likely that PPIX interferes with antibiotic efflux dependent on the MacB ABC transporter by an uncharacterized mechanism. This experimental observation prompted us to test whether PPIX could be expelled by this pump.

**Table 2:** Effect of PPIX overproduction or 5-ALA addition on erythromycin MIC of strain *ΔacrAB* overexpressing various efflux pumps.

	−5-ALA		+5-ALA
	−YfeX	+YfeX	−YfeX
JP313 (WT)	>32	>32	>32
*acrAB* (pBAD33)	1	1	1
*acrAB* (pBAD33-macAB)	>16	1	>16
*acrAB* (pBAD33-mdtEF)	>16	>16	>16
*acrAB* (pBAD33-emrAB)	>16	>16	>16
*acrAB* (pBAD33-acrEF)	>16	>16	>16
*acrAB* (pBAD33-acrAB)	>16	>16	>16

Erythromycin MIC in *μ*g mL^−1^ of strain *acrAB* overexpressing various efflux pumps. All strains were grown in LB medium + 0.2% arabinose to induce the efflux pump genes on pBAD33 and *yfeX* on pBAD24. Strains with YfeX overproduction (+YfeX) carry pBAD24-yfeX. Strains without YfeX (−YfeX) carry pBAD24 without insert. Strains without Yfex carrying pBAD24 without insert were also grown in LB + 0.2% arabinose with or without 5-ALA to overproduce COPIII. MIC was determined as described in Experimental Procedures.

### The *Escherichia coli* macAB mutant accumulates PPIX

To test whether MacAB is involved in PPIX efflux, the *ΔmacAB*::*Km* mutation was constructed and introduced into JP313(pBAD24-yfeX). The *macAB* mutant was studied as the *tolC* mutant (see the first result section). Upon arabinose induction, both strains produced similar amounts of YfeX, as determined by immunodetection (Fig. 1A). While both the WT and *macAB* mutant showed characteristic red porphyrin fluorescence under UV light, the fluorescence intensity of the *macAB* mutant was considerably higher compared to that of the WT, but lower than that of the *tolC* mutant (Fig. 1B). Again, complementation with pBAD33-macAB reduced the fluorescence to the level of the tolC+ strain (Fig. S1). HPLC analysis of the porphyrin content in the *ΔmacAB* mutant showed that the main peak in the cell extract was PPIX (Fig. 1C). Unlike the uninduced *tolC* (pBAD24-yfeX) mutant, the *macAB* (pBAD24-yfeX) mutant cells accumulated similar amounts of COPI and COPIII compared to the parental (pBAD24-yfeX) strain (data not shown). This raised the question as to whether COPIII might use the MacAB-TolC pump for efflux and whether there are other pumps functioning in the COPI and COPIII efflux. We first confirmed previously published results (Tatsumi and Wachi 2008) that the *macAB* mutant grown in the presence of 10 *μ*g mL^−1^ of 5-ALA did not accumulate porphyrins, indicating that MacAB is not the major COPIII efflux pump (data not shown). We also tested the effect of COPIII overproduction on MacAB-TolC macrolide efflux capacities. Strain *acrAB* (pBAD33-macAB) was grown in the presence of 10 *μ*g mL^−1^ 5-ALA plus or minus arabinose to induce the MacAB pump. Unlike PPIX accumulation, 5-ALA driven COPIII accumulation did not change the macrolide MIC (Table 2).

### PPIX accumulation in tolC and macAB mutants leads to increased photosensitivity

Augmentation of photosensitivity via forced overproduction of porphyrin heme intermediates and subsequent clearance of microbes by photodynamic therapy is an established antimicrobial treatment (Fotinos et al. 2008). We sought to determine whether bacteria overexpressing YfeX with an accumulation of PPIX were more sensitive to light irradiation. To elucidate this, we grew cells in the presence of arabinose to induce expression of the YfeX protein leading to accumulation of high levels of PPIX. We subsequently exposed these cells to light at a fluence rate of 1.375 mW/cm^2^ and examined cell survival by plating and counting the colonies. Bacterial survival was measured as the % of bacterial viable cells (CFU) after light irradiation compared to the CFU in dark. No toxicity was observed in the dark (data not shown) or in the absence of arabinose (Table 3). In the presence of light and *yfeX* induction, the number of CFU of all strains decreased. After irradiation with a light dose of 20 J/cm^2^, bacterial survival was 53% for the parental, 2% for the *tolC*, and 8% for the *macAB* mutant (Table 3). Thus, *E. coli* cells overproducing YfeX showed increased photosensitivity and correlated well with the accumulation of PPIX.

**Table 3:** Photosensitivity of various strains with or without YfeX overexproduction.

ara	Light dose (J/cm^2^)	0	5	10	15	20
WT	**−**	100	100	98	98	98
WT (pBAD24-yfeX)	**−**	100	90	90	100	130
	**+**	100	67	70	65	53
*tolC* (pBAD24-yfeX)	**−**	100	95	90	95	50
	**+**	100	70	55	8	2
*macAB* (pBAD24-yfeX)	**−**	100	100	115	125	130
	**+**	100	76	44	28	8

Bacterial survival was expressed as the % of bacterial viable cells (CFU) after light irradiation compared to the CFU in dark. Wild type and mutant cells did not show any CFU decrease when incubated in dark during the time course experiments. All experiments were done five times with standard deviations of 10% of the values indicated in the table. It is the maximal observed standard deviation. Cells were grown overnight in LB medium in the dark and 100-fold-diluted in the morning and kept in the dark. When they reached an OD_600_ of 0.2, 0.2% of arabinose was added. Cells were harvested still in the dark, when they reached an OD_600_ of 1. All bacterial cultures were diluted in PBS for the same OD_600_. This is considered time 0 in all the experiments.

### PPIX accumulation in iron-chelated tolC and macAB mutants

To test whether inhibition of iron insertion into PPIX, which is the last step in heme biosynthesis, leads to PPIX accumulation, we used an *E. coli* strain *entF* lacking the major siderophore enterobactin. Enterobactin is able to bind and solubilize iron Fe^+++^and thus to uptake the iron–enterobactin complex through specific outer membrane receptors and permeases, reducing the bacteria iron starvation. Thus, *E. coli* strain *entF* mutant is easier to starve of iron. Strains *entF*, *entF macAB*, and *entF tolC* were grown as described in Experimental Procedures in the presence of the iron chelator 2-2, dipyridyl (Dip) (0, 50 or 100 *μ*mol/L). The *entF* and the isogenic mutants *entF tolC* and *entF macAB* had the same growth rate in LB Dip + 50 *μ*mol/L and 100 *μ*mol/L and stopped growing at 200 *μ*mol/L or greater concentrations, indicating that the three strains have similar sensitivity to iron chelation by Dip (data not shown).

**Figure 2:**
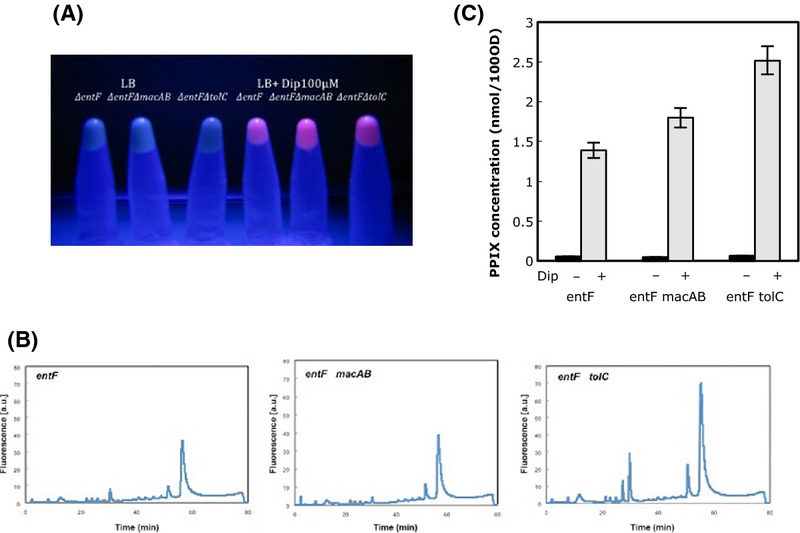
PPIX accumulation in various strains grown with or without an iron chelator (Dip). Cells were grown overnight in LB medium and 100- 11-fold-diluted in the morning. When they reached an OD_600_ of 0.5, Dip was added to half of the cultures at the final concentration of 100 *μ*mol/L. Cells were harvested, when growth stopped for the cultures grown in the presence of Dip and bacterial cultures were diluted to the same OD_600_ in PBS. (A) Visualization of fluorescence of the soluble fraction under near-UV light. Name of the strains and addition of iron chelator (Dip) are indicated on (A). All experiments were done five times. (B) HPLC analysis of the porphyrin content in the three iron-chelated cultures prepared as described above. The main peak corresponds to PPIX. (C) PPIX concentration in cells grown with or without iron chelator. The same cultures grown with and without Dip were harvested, diluted to the same OD_600_, and total intracellular porphyrin concentrations were determined by fluorescence spectrometry with a near-UV an excitation. Porphyrin concentrations were normalized so as to represent the porphyrin amount in cell lysates corresponding to a bacterial suspension with an optical density of 100 at 600 nm (OD_600_ 100). The error bars represent the standard deviation for three experiments.

In the absence of Dip, cultures were non-fluorescent under near-UV light. In the presence of the iron chelator, the *entF* strain was slightly fluorescent whereas, *entF tolC* and *entF macAB* showed strong fluorescence (Fig. 2A). The total porphyrin intracellular contents were determined by fluorescence spectrometry with a near-UV excitation (Fig. 2C). The total intracellular porphyrins in the *entF* increased 10 times in the presence of iron chelators as compared to the Dip free medium. This tendency was even more pronounced for the *entF macAB* and *entF tolC* strains where a boost of 20 and 25, respectively, was achieved in the absence of iron. HPLC analysis of the porphyrin content in the three iron-chelated cultures confirmed these results and showed that the main compound in the cell extract corresponds to PPIX (Fig. 2B). Therefore, in the absence of intracellular iron to ligate PPIX, there was an accumulation of porphyrins including PPIX which is expelled by the MacAB-TolC pump.

Iron lacking cultures when irradiated with light also showed a significant reduction in survival. Under these conditions and a light dose of 10 J/cm^2^, the survival of the *entF* strain was 20%, which dropped to 0.5% for the *entF macAB*, and to 0.002% for the *entF tolC* strain (Table 4).

**Table 4:** Photosensitivity of various strains in iron-chelated medium.

Dip	Light dose (J/cm^2^)	Dark	Light		
		0	0	5	10
WT (*EntF+*)	**−**	100	100	100	100
	**+**	100	100	100	100
*entF*	**−**	100	100	98	45
	**+**	100	100	50	20
*entF tolC*	**−**	100	100	77	40
	**+**	100	100	0.006	0.002
*entF macAB*	**−**	100	100	100	45
	**+**	100	100	2	0.5

Bacterial survival was expressed as the % of bacterial viable cells (CFU) after light irradiation compared to the CFU in dark. Wild type and mutant cells did not show any CFU decrease when incubated in dark during the 2 h experiments. Survival was measured at time point 0; 5 and 10 J/cm^2^. All experiments were done five times with standard deviations of 10% of the values indicated in the table. It is the maximal observed standard deviation. Cells were grown overnight in LB medium in the dark and 100-fold-diluted in the morning and kept in the dark. When they reached an OD_600_ of 0.5, Dip was added at the final concentration of 100 *μ*mol/L. Cells were harvested still in the dark, when growth stopped and bacterial cultures were diluted in PBS for the same OD_600_. This is time 0 of the experiments.

## Discussion

In the present study, we used an *E. coli* strain overexpressing YfeX, leading to increased PPIX formation from endogenous heme, to search for multidrug pump(s) involved in PPIX efflux. We showed that a *tolC* mutant accumulated high amounts of PPIX. Since TolC is the outer membrane component of tripartite efflux pumps, this strongly suggested that PPIX was effluxed from cells through the TolC channel. However, the inner membrane pumps presumably involved in this process were unknown. We chose to examine five *E. coli* efflux pumps belonging to MDR families found in Gram-negative bacteria: three of them, AcrAB, MdtEF, and AcrEF, belong to the RND family; one, MdtEF, to the MF family; and one, MacAB, to the ABC family (Nishino et al. 2009). While AcrAB is the major multidrug efflux pump in *E. coli*, the others do not contribute to antibiotic intrinsic resistance. However, all five pumps increased antibiotic resistance when they were overexpressed on plasmids (Nishino and Yamaguchi 2001; Nishino et al. 2009). Interestingly, we noted that YfeX-driven PPIX overproduction decreased the ERY resistance resulting from MacAB plasmid overproduction, but did not affect the antibiotic MIC of the other tested pumps. The fact that PPIX overproduction did not affect the MICs of strains overexpressing other tested efflux pumps suggested that PPIX did not sensitize cells or lead to a general blockage in drug efflux. Instead, our results lead to the hypothesis of a competition between PPIX and macrolides at the level of the MacAB efflux pump. In general, however, substrate competition for efflux does not occur in bacteria or mammalian P-glycoprotein pumps, suggesting that drugs have distinct substrate binding sites on the ABC component of these pumps (Elkins and Mullis 2007). Despite this, the specific inhibition of the MacAB pump by PPIX prompted us to test whether MacAB is involved in PPIX efflux.

The *tolC* and *macAB* mutants accumulate more PPIX than the parental isogenic strain when either *yfeX* was induced or when cells void of iron. Our results point to MacAB as a major inner membrane component for PPIX efflux. The fact that the *tolC* mutant accumulated more PPIX than the *macAB* mutant suggests that other inner membrane components might form minor tripartite PPIX efflux pumps with TolC. Tatsumi and Wachi (2008) found that the WT and the *tolC* mutant mainly formed COPIII (>94% of total intra cellular porphyrins) but not PPIX when bacteria were supplemented with 5-ALA. The *tolC* mutant accumulating COPIII, indicates that COPIII is also excluded from cells by efflux. However, mutations in genes encoding several MDR pumps including *macAB* did not increase COPIII accumulation (Tatsumi and Wachi 2008). We reproduced these results in our *macAB* mutant. Both macrolides and PPIX are very hydrophobic molecules, whereas COPIII with side chains containing 4 propionic acids is much less hydrophobic (Ding et al. 2001). This could result in differing pump specificities. It is also possible that COPIII is expelled by several pumps or by a yet unidentified pump.

PPIX efflux pumps were characterized for the first time in *Streptococcus agalactiae* (Fernandez et al. 2010). This heme auxotroph pathogen uses exogenous heme for respiration and has two efflux pumps, PefAB and PefCD, responsible for heme and PPIX efflux. PefAB belongs to the MFS family, whereas PefCD belongs to the ABC family. In *S. aureus,* it was shown that the ABC pump HtrAB might be involved in the export of unidentified toxic compounds which accumulate when cells are grown in the presence of heme (Stauff et al. 2008). *Staphylococcus epidermidis* might have a similar system (Juarez-Verdayes et al. 2012). In human cancer cells in culture, it was also shown that the ABCG2 ABC pump was responsible for PPIX export across the plasma membrane (Ogino et al. 2011).

Several lines of evidence suggest the existence of a link between heme or PPIX levels and bacterial virulence. A *S. agalactiae pefR* mutant with increased expression of PPIX and heme efflux pumps is attenuated for virulence (Fernandez et al. 2010). On the other hand, *S. aureus hssS-hssR* mutants unable to induce HtrAB, the heme efflux pump, are hypervirulent, and it was proposed that increased intracellular heme concentrations in these *S. aureus* mutants could induce stress that would lead to elevated secretion of virulence factors (Torres et al. 2007). A *Salmonella typhimurium* strain lacking several drug efflux systems was more sensitive to various antibiotics and avirulent when mice were inoculated by the oral route, and deletion of the *macAB* genes attenuated *Salmonella* virulence (Nishino et al. 2006). These results suggested that the MacAB pump was one of the major factors contributing to bacterial virulence.

Recently, it was shown that the *macAB* efflux pump is required for survival of *Salmonella* in the inflamed intestines as well as in macrophages, where these organisms are exposed to highly toxic ROS (Bogomolnaya et al. 2013). The MacAB pump was not required for survival in macrophages deficient for ROS production. It is well established that porphyrin decomposition increases the photogeneration of peroxide (Kochevar 1987; Komagoe and Katsu 2006). It is tempting to propose that in *S. typhimurium,* the MacAB pump is involved in the efflux of intracellular PPIX which decreases ROS formation in the bacteria cytoplasm.

In mammalian cancer cells, inhibition of ferrochelatase by iron chelators resulted in a strong increase in the intracellular PPIX pool in cells in the absence or presence of 5-ALA supplementation, leading to an increase in photodynamic sensitivity (Blake et al. 2011). Here, we showed that iron-depleted cultures of *tolC* and *macAB* mutants accumulated increased amounts of PPIX and were dramatically more sensitive to photodynamic action. This was not the case for the WT strain grown in the iron-depleted medium, indicating that the intracellular PPIX formed in iron-chelated medium could still be expelled by the MacAB-TolC pump.

Photodynamic therapy using 5-ALA as a precursor of the photosensitizer porphyrins is used to kill microorganisms mostly in localized superficial infections. Our work indicates that inhibition of multidrug efflux pumps belonging to the ABC family together with an iron-chelating agent could strongly potentiate the protoporphyrin-IX-mediated photodynamic inactivation of microorganisms.

Deferrochelatases are widespread among Gram-positive and -negative bacteria. In many species, their structural genes are regulated by the Fur repressor and are induced in iron-depleted media. Thus, addition of an iron chelator could enhance photodynamic therapy even in the absence of 5-ALA.

In conclusion, we propose that PPIX is an endogenous substrate of the MacAB-TolC pump in *E. coli* and *S. typhimurium* and that this compound is produced inside bacteria when natural heme homeostasis is disrupted by the iron shortage occurring when bacteria invade the mammalian host.

As a working hypothesis, we propose that inside host macrophages, the *S. typhimurium* MacAB-TolC pump expels the formed PPIX from the bacteria decreasing ROS formation, providing a possible explanation for the role of MacAB in *S. typhimurium* pathogenicity.

## Supporting Information

Figure S1 Visualization of fluorescence under near-UV light of the soluble fractions of strains producing YfeX. Relevant mutations and plasmids present in the strains are indicated above the figure. Wild type (WT), *macAB* and *tolC* mutants showed red porphyrin fluorescence under UV light. Complementation of the *macAB* and t*olC* mutations with pBAD33-macAB or pACYC184-tolC, respectively, reduced the fluorescence to the level of WT strain.

Figure S2 Impact of the arabinose-inducible expression of MacAB and YfeX onto the drug resistance phenotype of JP313*ΔacrAB*. The disk diffusion susceptibility test consisted of spreading a 100-fold-diluted overnight LB culture onto Mueller–Hinton agar medium supplemented with arabinose. Drugs were: erythromycin (ERY); trimethoprim (TMP); azithromycin (AZI); desoxycholate (DOC); novobiocin (NOV); kanamycin (KAN); Ampicillin (AMP); chloramphenicol (CMP). (A) *acrAB* (pBAD33); (B) *acrAB* (pBAD33-macAB); (C) *ΔacrAB* (pBAD33-macAB)(pBAD24-yfeX).
