# Supplementary figures and images for: Protoporphyrin (PPIX) efflux by the MacAB-TolC pump in Escherichia coli
Source: Microbiologyopen. 2014 Sep 26;3(6):849–59. doi: 10.1002/mbo3.203 (PMC4263509; doi:10.1002/mbo3.203)

Figure S1

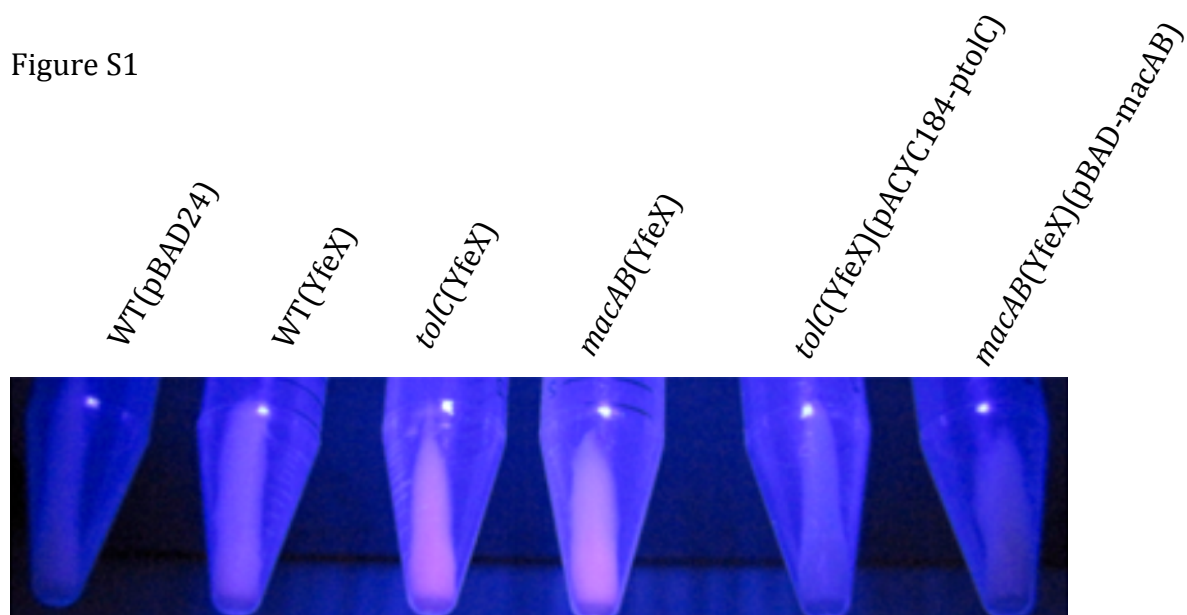

Supplement: Figure S1 — Visualization of fluorescence under near-UV light of the soluble fractions of strains producing YfeX. Relevant mutations and plasmids present in the strains are indicated above the figure. Wild type (WT), macAB and tolC mutants showed red porphyrin fluorescence under UV light. Complementation of the macAB and tolC mutations with pBAD33-macAB or pACYC184-tolC, respectively, reduced the fluorescence to the level of WT strain. [file mbo30003-0849-sd1.pdf]

Fig. S2

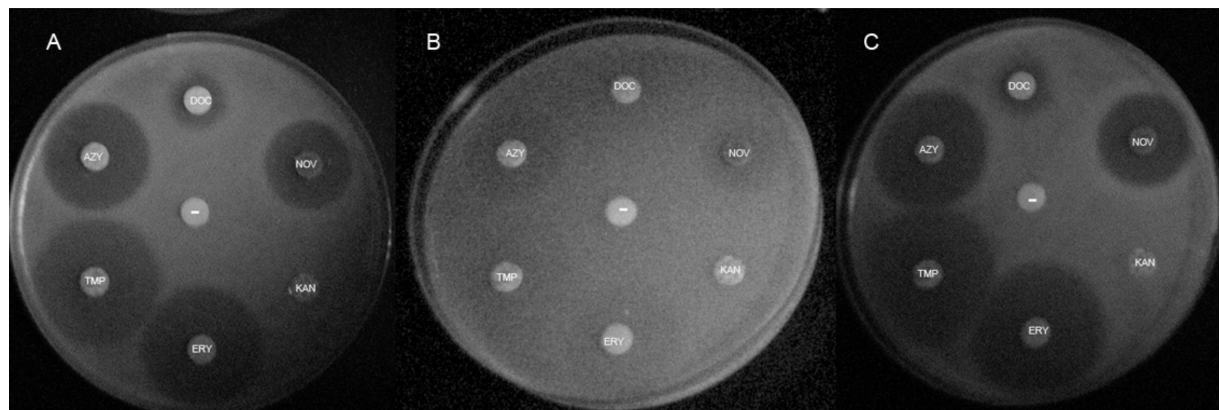

Supplement: Figure S2 — Impact of the arabinose-inducible expression of MacAB and YfeX onto the drug resistance phenotype of JP313ΔacrAB. The disk diffusion susceptibility test consisted of spreading a 100-fold-diluted overnight LB culture onto Mueller–Hinton agar medium supplemented with arabinose. Drugs were: erythromycin (ERY); trimethoprim (TMP); azithromycin (AZI); desoxycholate (DOC); novobiocin (NOV); kanamycin (KAN); Ampicillin (AMP); chloramphenicol (CMP). (A) acrAB (pBAD33); (B) acrAB (pBAD33-macAB); (C) ΔacrAB (pBAD33-macAB)(pBAD24-yfeX). [file mbo30003-0849-sd2.pdf]
